# Impatient Science

**Published:** 1892-04-16

**Authors:** 


### The Doctor's Armchair.

### IMPATIENT SCIENCE.

"Art is long and life is fleeting." The man who has knowledge in his possession, and a strong conviction of the value of that knowledge to his fellow-men, is often surprised and sometimes made angry by the resistance of the unthinking and the opposition of the wrong-thinking to the practical utilisation of his knowledge on the largest possible scale. A writer in a medical contemporary has allowed his equanimity to be disturbed by the anti-vaccinators, the anti-vivisectors, and what he describes as the "anti-party" generally. This gentleman permits himself the use of strong language. "Faddists" he calls his unscientific opponents, and he says that their "mischievous' activity is provocative of "irritation and disgust." They have much "to answer for," he considers. "They arrest progress, they appeal to ignorant prejudice, they frequently resort to plausible misrepresentations, they are possessed with a fanatical enthusiasm, they fill the papers with their cries and declamations, and their statements rarely include the whole truth."

Coming from a man of scientific training, whose passions, one may suppose, have long been in the habit of obeying the curb of calm reason and high philosophy, this language strikes one as being decidedly energetic. From the point of view of the ordinary man it will not be considered any the worse for that; but it certainly is not, or, at least, it ought not to be, the language of the laboratory. The business of science is to discover facts, weigh and measure facts, collate facts, reason about facts, and generally to make up facts into appropriate parcels, and label them neatly for the shelves of the scientific warehouse. But to stand at the door of that warehouse with arms akimbo, and to vituperate at large against an ignorant, uncomprehending and obstructive *canaille*—this is surely a work which a dignified science cannot but frown upon.

It seems to us that the anti-vaccinators are, as a class, persons of much enthusiasm, but little knowledge; and the anti-vivisectors appear to be in the same position. But is not this description applicable to the unscientific world generally? Science does not live in a world by itself; it lives among ordinary people; and the scientists were, for the most part, ordinary people, and the children of ordinary people, before they were made free of the mysteries of the laboratory. Science, it is to be observed, is, as we understand it, a comparatively new thing in the world. It is by no means the first new thing. New things, in this unphilosophic part of creation, have generally an experience peculiar to themselves. Or rather their experience may not be so peculiar as it seems. New men and new things in new circumstances often receive scant courtesy. They are watched by established men as strangers are by suspicious house-dogs. They have to establish a claim to recognition, and to a place among their equals, by proving their possession of actual worth.

Science is now claiming to be a practical leader of men. Mr. George Meredith thinks she has shown us all she has to show, and that on the whole she is not very fit to be a leader. We do not quite agree with him, but it is pretty certain that she will never be a leader if she be a persistent vituperator. History has not been written for nothing. Science may well take a leaf out of the book of religious history. In the early days of religions their founders and first disciples have shown their greatness, not by their success in scolding, but by their extraordinary capacity for living religious lives and teaching religious doctrines. The scientific man may think that science is much more important than religion, that it appeals much more immediately to the reason, and that therefore it ought to be promptly and universally accepted by reasonable creatures like men and women. But it is probable that almost a majority of mankind will dispute every one of these premises. The religious man puts religion decidedly in front of science in his estimation, and the politician but too often regards the denizen of the laboratory as a bespectacled, snuffy-coated, and unpractical faddist. It is important to recollect in this connection what Carlyle said: "It is not this man, nor that man, but all men who make up mankind, and their united tasks the task of mankind."

Vaccination and vivisection are questions of immediately practical moment both to physiologists and to people in general. It is to be admitted, on the one hand, that physiologists know a great deal about these questions, and people in general but a very little. The rational presumption is that the physiologists are right. But, even if they are, their science is no more to be crammed down the public throat by force than the religion of the religionists is to be made to prevail by the same process. There have been races that have refused religion, and others that have thrown it off after having once accepted it. A philosophic consideration of things would lead us to anticipate that here and there science may meet with precisely the same experience. It is not every man who is able to take a rational and scientific view of things, and if there are some who are unable, there are probably more who are unwilling even to make the attempt. There are only two possible ways of propagating scientific ideas in the world—the one is by persuasion, the other by force. Is there anybody who would seriously propose to propagate science by legal enactment and judicial penalty? But if scientific ideas are not to be propagated by force, wherein lies the fitness of vituperation as a method of propagation? The man who would vituperate would compel if he could. Vituperation is, in its essence, a form of persecution. Persecution never did serve the purpose of the persecutor in the long run, and never will.

It is a curious commentary on the popular theory of a steadily progressive world that the old-fashioned weapons which a too eager religion has been compelled to lay down are looked at wistfully and handled approvingly by a science which is eager to make itself universally felt. The motive of the scientific propagandist is beyond all praise, but his method, when he takes to the vituperation of his opponent is worthy of nothing but reprobation. Nothing is more dishonouring to true science than to arm her with the clubs and brickbats beloved of crude impatience, or with the statutes and penalties preferred by narrower, even if astuter, minds. Science must win her way by teaching and persuasion alone. When she keeps to this method she is invariably successful. When, as she has sometimes done, she has borrowed the wicked weapons of the persecutor, she has degraded herself, and played the game of the enemy.

